# The Kirkendall effect and nanoscience: hollow nanospheres and nanotubes

**DOI:** 10.3762/bjnano.6.139

**Published:** 2015-06-18

**Authors:** Abdel-Aziz El Mel, Ryusuke Nakamura, Carla Bittencourt

**Affiliations:** 1 Institut des Matériaux Jean Rouxel, IMN, Université de Nantes, CNRS, 2 rue de la Houssinière, BP 32229, 44322 Nantes cedex 3, France; 2 Department of Materials Science, Graduate School of Engineering, Osaka Prefecture University, Gakuen-cho 1-1, Naka-ku, Sakai 599-8531, Japan; 3 Chimie des Interactions Plasma-Surface (ChIPS), CIRMAP, Research Institute for Materials Science and Engineering, University of Mons, 23 Place du Parc, B-7000 Mons, Belgium

**Keywords:** hollow nanospheres, Kirkendall effect, metals, nanotubes, oxides

## Abstract

Hollow nanostructures are ranked among the top materials for applications in various modern technological areas including energy storage devices, catalyst, optics and sensors. The last years have witnessed increasing interest in the Kirkendall effect as a versatile route to fabricate hollow nanostructures with different shapes, compositions and functionalities. Although the conversion chemistry of nanostructures from solid to hollow has reached a very advanced maturity, there is still much to be discovered and learned on this effect. Here, the recent progress on the use of the Kirkendall effect to synthesize hollow nanospheres and nanotubes is reviewed with a special emphasis on the fundamental mechanisms occurring during such a conversion process. The discussion includes the oxidation of metal nanostructures (i.e., nanospheres and nanowires), which is an important process involving the Kirkendall effect. For nanospheres, the symmetrical and the asymmetrical mechanisms are both reviewed and compared on the basis of recent reports in the literature. For nanotubes, in addition to a summary of the conversion processes, the unusual effects observed in some particular cases (e.g., formation of segmented or bamboo-like nanotubes) are summarized and discussed. Finally, we conclude with a summary, where the prospective future direction of this research field is discussed.

## Review

### Introduction

**Figure 1:**
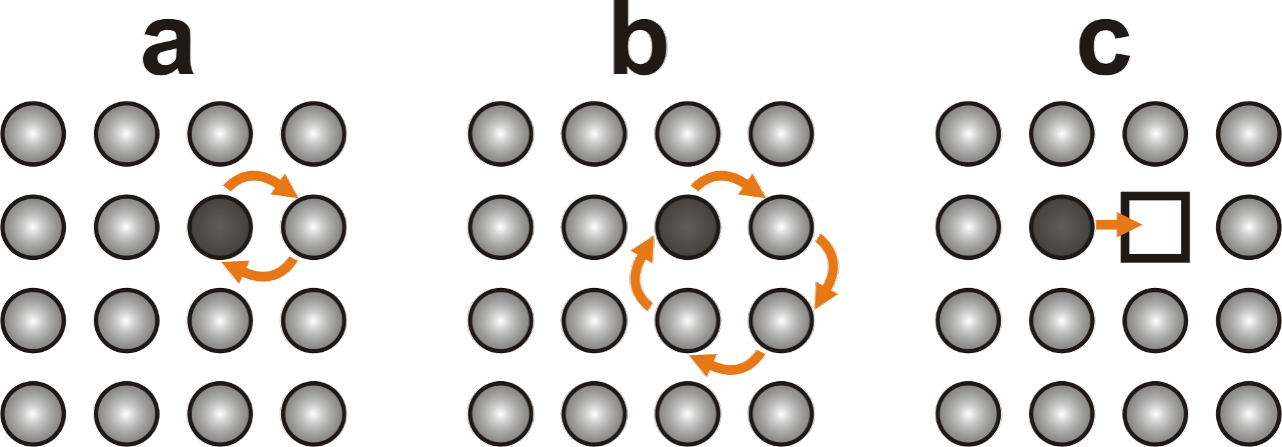
Atomic diffusion based on (a) the direct exchange mechanism, (b) the ring mechanism and (c) the vacancy mechanism. Figure adapted with permission from [3], copyright 1997 Springer Science and Business Media.

In the years following the discovery of the diffusion of gold in solid lead by Roberts-Austen in 1896 [1–2], it was believed that atomic diffusion in metals could occur by a direct exchange of atomic position (Figure 1a) or by a ring mechanism in which the atoms exchange their positions by following a ring path (Figure 1b) [3]. However, in 1942, Kirkendall reported on a new diffusion phenomenon, known today as the Kirkendall effect, explaining the interdiffusion between copper and zinc in a copper/brass system [3–4]. The experimental data reported by Kirkendall supported the theory that atomic interdiffusion at the interface of two metals occurs through a vacancy exchange mechanism (Figure 1c). Despite the high importance of this discovery, at that time, it did not receive much attention [3]. In April 1946, together with his student Smigelskas, Kirkendall submitted a manuscript to the editorial office of Transactions of the AIME describing new results supporting the theory he had proposed four years before [3]. Unfortunately, this manuscript was rejected by the referee (R. F. Mehl from the Carnegie Institute of Technology) who was convinced that the new diffusion mechanism proposed by Kirkendall was wrong. In 1947, Smigelskas and Kirkendall succeeded in publishing their article [5] after including the criticisms and comments of Mehl in the comments and discussion section [3]. In 1950, Mehl acknowledged the validity of the Kirkendall effect [3] and one year later he and his student DaSilva published new data validating the reproducibility of the Kirkendall effect for different metal alloys [6].

**Figure 2:**
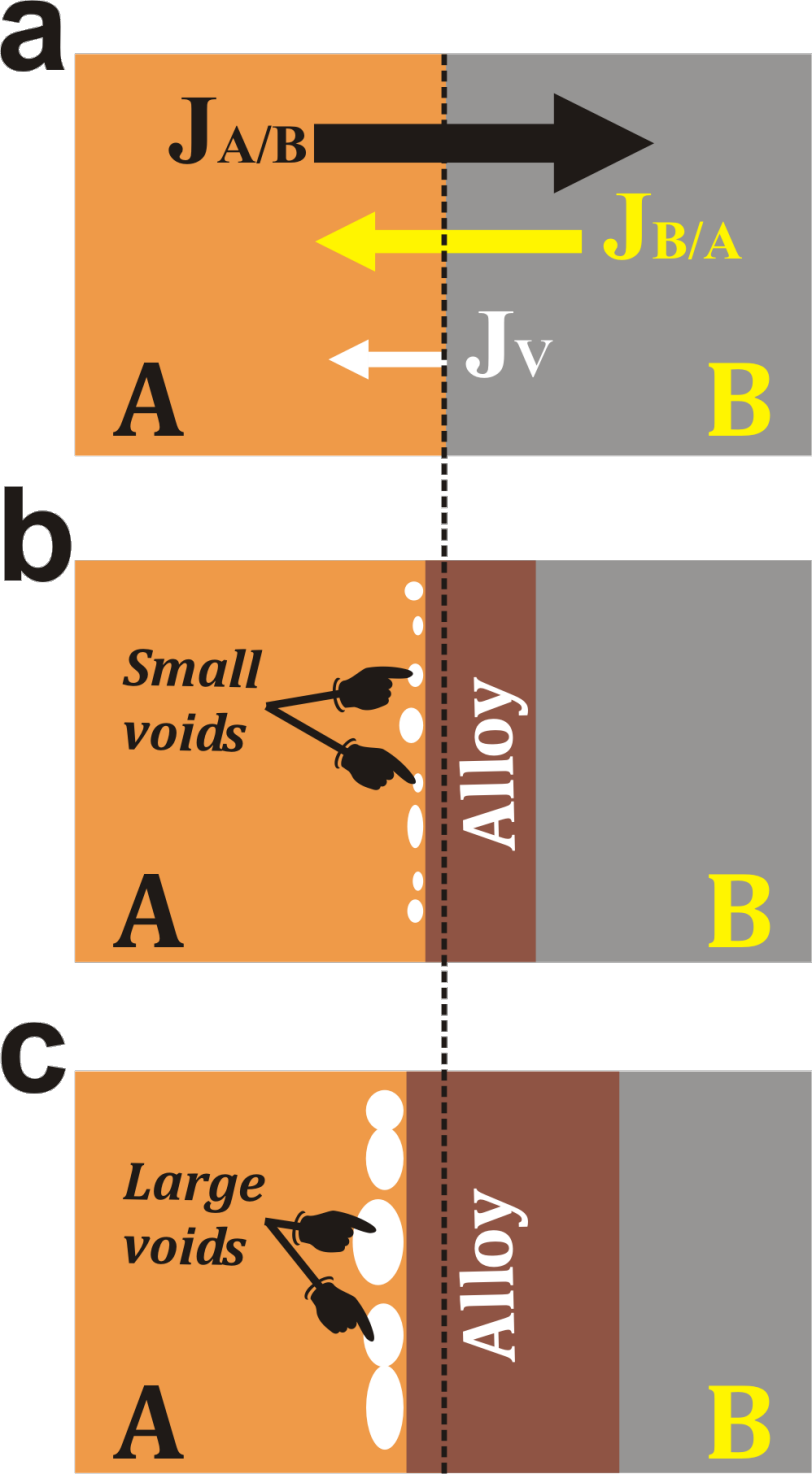
Schematic illustration describing the different stages occurring during the formation of voids at the interface of two different metals, A and B, as a consequence of the Kirkendall effect induced by thermal annealing. (a) Two stacked metals A and B before annealing; the arrows describe the flux of the different species diffusing from metal A to B and vice versa. Formation of small (b) and large (c) voids within metal A, as a consequence of the unbalanced diffusion rate.

The Kirkendall effect describes the motion of the boundary between two metals due to a thermally activated, unbalanced diffusion. Upon annealing of two stacked metals, A and B, at a temperature high enough to thermally activate the diffusion of atoms, atomic migration can occur at the interface where atoms diffuse from metal A to metal B and vice versa (Figure 2a). Such an annealing process results in the formation of an A/B alloy layer located between the two sides of the interface where the final thickness is dependent on both the annealing temperature and time. According to the Kirkendall effect, the position of the initial interface changes during the annealing process since the atomic diffusion coefficients of atom A in metal B and of atom B in metal A are different. As a consequence of the unbalanced diffusion rates between the two stacked metals, vacancies will be injected at the interface region within the faster diffusing metal. For example, if we consider that atom A diffuses in metal B much faster than atom B in metal A, the flux of atoms migrating from metal A to metal B (*J*_A/B_) will be much higher than atoms of B diffusing in the opposite direction (*J*_B/A_). In such case, the A/B alloy region will be more extended within metal B and vacancies will be injected at the interface region within metal A (Figure 2b). The coalescence of excess of vacancies leads to the formation of small voids distributed all along the interface. As the annealing process progresses in time, vacancies will be generated leading to the enlargement of the formed voids (Figure 2c) that will coalesce and form pores within the material.

Until recently, the formation of pores in metal alloys as a consequence of the Kirkendall effect was considered as a serious problem in metallurgy. For example, porosity at the interface of two soldered metals deteriorates the mechanical properties of the interface, resulting in a reduced mutual adhesion between them. For this reason, the main purpose of the research carried out in the past on the Kirkendall effect was to develop technological solutions to avoid the mentioned drawbacks, which lead to the failure of interfaces between soldered metals. However, in 2004, Yin et al. brought the Kirkendall effect to light when they demonstrated for the first time that at the nanoscale, this effect becomes an extremely useful “tool” for the engineering and design of hollow nanomaterials [7]. Since this discovery, the Kirkendall effect applied to nano-objects has been considered as a very powerful synthesis strategy for hollow nanomaterials. The importance here rises from the fact that hollow nanostructures find applications in various technological applications including biotechnology [8], batteries [9–13], sensors [14–16], catalysis [17–19], photodetectors [20], electrochromic devices [21] and supercapacitors [22]. Three years after the pioneering work of Yin et al. [7], the progress in the synthesis of nanotubes and hollow nanoparticles using the Kirkendall effect was reviewed by Fan et al. [23]. In this first review, they summarized and discussed the achievements from both the experimental and theoretical point of view. In 2012, a review focusing mainly on the synthesis of hollow nanospheres using the Kirkendall effect was reported by Wang et al. [24]. While this present review was in preparation, Anderson and Tracy published a review on the progress of different synthesis approaches of hollow nanoparticles, including the anion and galvanic exchange reaction and the Kirkendall effect [25].

This review has two main goals: (i) expanding the knowledge on hollow nanospheres by addressing some issues which were not taken into account in the previous reviews (e.g., dynamic transformation of nanospheres from solid to hollow) and (ii) providing an updated account on the recent progress in the synthesis of nanotubes using the Kirkendall effect. We begin by summarizing the state of the art on the synthesis of Kirkendall hollow nanospheres with a special emphasis on the conversion mechanisms (i.e., symmetric and asymmetric) which require, in some cases, in situ dynamic analysis to be understood. We follow with a brief summary on the growth strategies developed so far to synthesize nanotubes using the Kirkendall effect. After, we mainly focus on the Kirkendall oxidation process of metal nanowires with special attention to some unusual effects observed for Kirkendall nanotubes which, until today, remain unclear.

### Hollow nanospheres

#### First observation: symmetrical conversion mechanism

The formation of hollow Kirkendall nanospheres was first reported by Yin et al. in 2004 [7]. They observed the formation of hollow nanospheres while exploring the sulfidation of cobalt nanoparticles by injecting a solution of sulfur in 1,2-dichlorobenzene into hot Co nanocrystals. They demonstrated that both the annealing temperature and the annealing time have a direct impact on the transformation kinetics of the nanoparticles from solid Co into hollow CoS. In this same study, the authors show that a similar effect occurs during oxidation or selenization of Co nanocrystals. Since this first report, the nanoscale Kirkendall effect has become very popular and been adopted by many research groups for the synthesis of hollow nanoparticles covering a wide range of materials including sulfides [8,26–30], oxides [9–11,30–52], selenides [30,42,53], telurides [14,30], fluorides [53–54], phosphides [17,55] and metals [56–59]. Through these studies, two conversion mechanisms were identified: symmetrical and asymmetrical. In this subsection, we focus on the symmetrical mechanism and the following we discuss the asymmetrical mechanism. The terms “symmetrical” and “asymmetrical” are in general employed to indicate whether the shell of the hollow nanosphere is isotropic or nonuniform, respectively.

**Figure 3:**
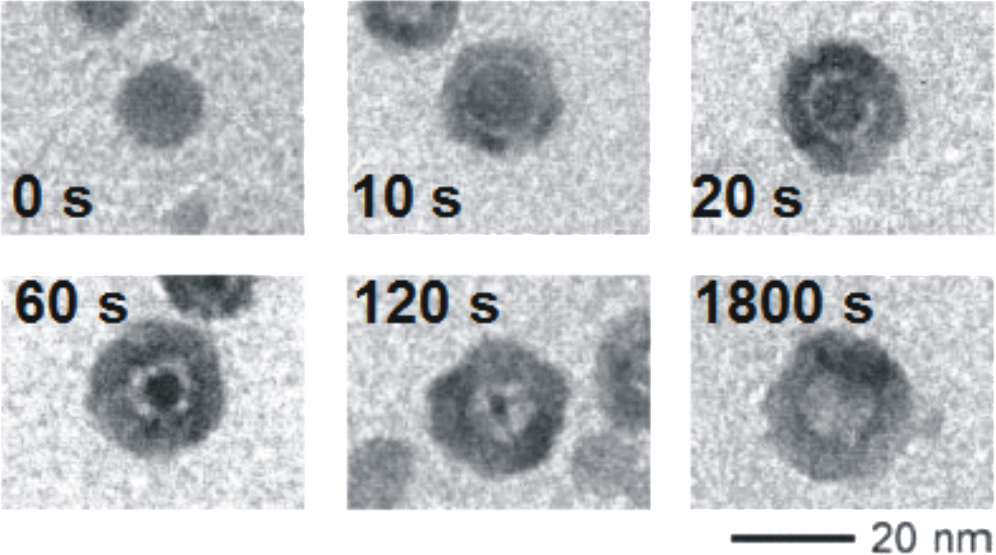
Morphological evolution of CoSe hollow nanocrystals as a function of the selenization time. Figure adapted with permission from [7], copyright 2004 American Association for the Advancement of Science.

**Figure 4:**
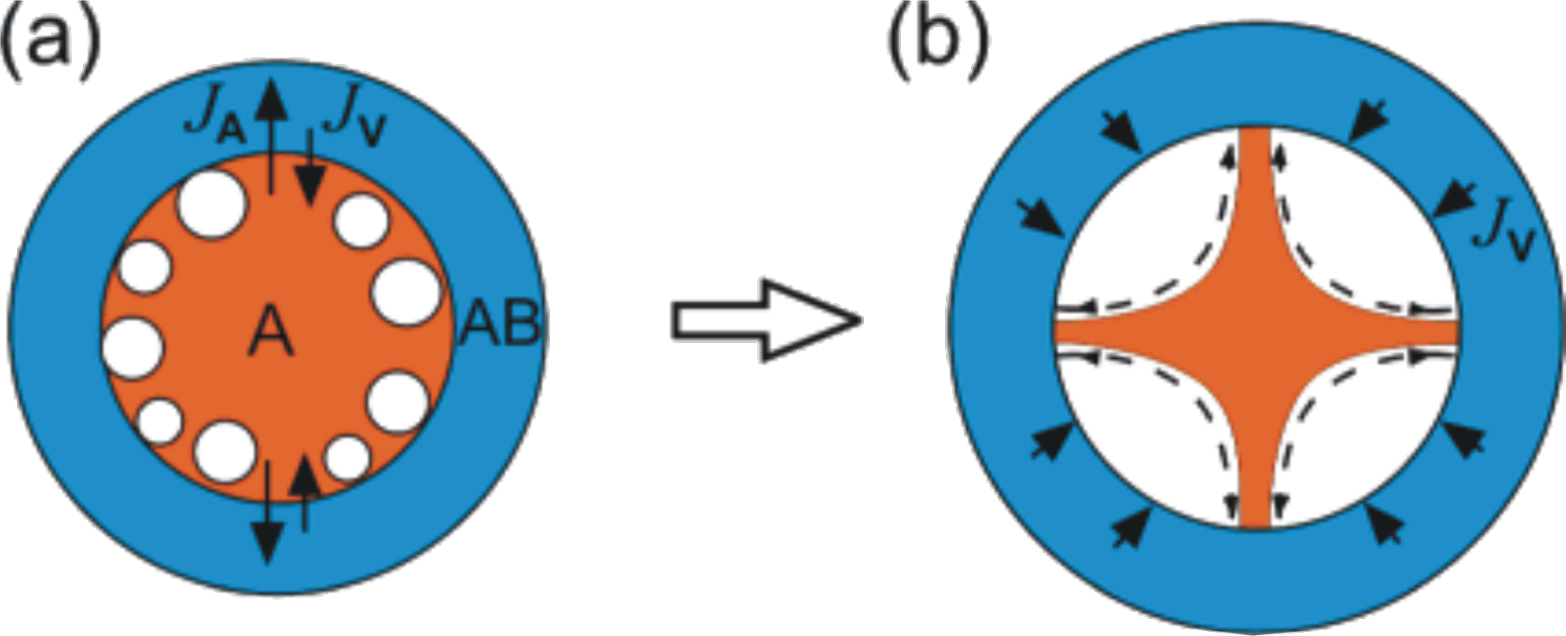
A scheme describing the Kirkendall-induced hollowing process of nanospheres consisting of a core/shell structure. (a) Generation of small Kirkendall voids along the core/shell interface and (b) surface migration of atoms along the bridges linking the core A to the shell AB. Figure adapted with permission from [61], copyright 2007 American Chemical Society.

An early example of the symmetrical conversion mechanism is the pioneering work of Yin et al. on the selenization of cobalt nanoparticles [7]. They have shown that the conversion reaction starts by the formation of a very thin cobalt selenide shell on the outer skin of the Co nanoparticle (Figure 3). As the reaction proceeds in time, the Co atoms tend to diffuse outward through the cobalt selenide shell until reaching the outer surface of the nanoparticle. Simultaneously, the Se atoms were reported to diffuse inward through the shell until reaching the Co core. Due to the high outward diffusion flux of Co compared to the slower inward flux of Se, vacancies are created and injected at the Co(core)/CoSe(shell) interface. The migration and agglomeration of vacancies result in the formation and merging of the initial voids located at the interface and extended along the Co core (Figure 4a). The increase in size of these voids was found to lead to the formation of Co bridges, linking the Co core and the CoSe shell (Figure 4b). The authors found that these bridges persist until the Co core is completely consumed during the reaction and the particle becomes fully hollow. Such bridges were also reported for other materials; it is believed that at this stage the metal atoms leaving the core are transported by diffusing mainly on the surface of the voids along the bridges until reaching the inner surface of the shell where they spread before diffusing outward across it [7,23,29,60–61]. Since the surface diffusion coefficients are several orders of magnitude higher than the bulk diffusion coefficients, at this stage, the growth kinetics of voids was reported to be faster than in the early stage of the growth were the formation of voids is mainly governed by a bulk diffusion mechanism [23,61].

#### Asymmetrical conversion mechanism

**Figure 5:**
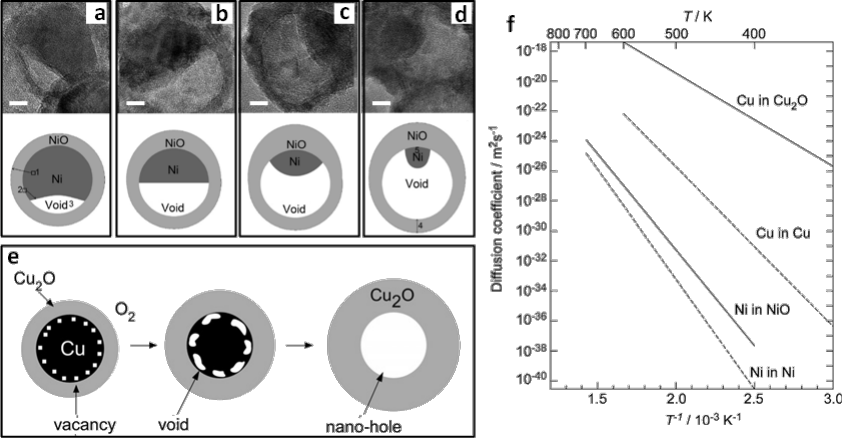
TEM micrographs and corresponding schemes of 26 nm nickel nanospheres oxidized in air at 300 °C for (a) 120, (b) 150, (c) 180, and (d) 210 min. Scale bar: 5 nm. Figure adapted with permission from [43], copyright 2010 American Chemical Society. (e) Scheme of the oxidation process of a Cu nanoparticle. (f) Self-diffusion coefficients of Ni and Cu and diffusion coefficients of Ni and Cu ions in NiO and Cu_2_O, respectively. Figure adapted with permission from [32], copyright 2008 Taylor & Francis.

The stages of the hollowing process previously described were found to vary according to the selected metal and the reactive atmosphere [25,27–28,43]. For example, when oxidizing nickel nanospheres under ambient atmosphere, Nakamura et al. found that the shape of the resulting nickel oxide hollow nanoparticles was asymmetric with a nonuniform shell thickness [32]. This effect was also observed recently by other groups [25,43,48]. On the basis of these studies, one can briefly summarize the stages of the asymmetric conversion mechanism. In the early stage of conversion, it is believed that the vacancies migrate and then agglomerate into a single void at the Ni/NiO interface on one side of the nanoparticle (Figure 5a–d) instead of being distributed along the metal/metal oxide interface as observed in the case of the symmetrical conversion mechanism during the oxidation of Cu nanospheres (Figure 5e). As the oxidation process proceeds in time, the growth of NiO was reported to occur preferentially at the adjacent side of the void (Figure 5b–d). In general, most authors report that the shell is thinner on the side where a large void is present during oxidation and thicker on the opposite side (Figure 5d). The formation of a single large void during the conversion process was assumed to be more favorable as compared to the formation of multiple smaller voids and filaments observed in the symmetrical mechanism, illustrated in Figure 4 and Figure 5e. Indeed, since the sum of surface energies of multiple small voids is higher than the surface energy of a single large void, during the conversion process, multiple small voids tend to agglomerate and form a single large void to minimize the surface energy [32].

Until today, the explanation of such asymmetrical conversion mechanism is still under discussion. In their early report, Nakamura et al*.* concluded that the formation of an off-centered, single void during oxidation of nickel is related to the intrinsic properties of nickel itself [32]. They came to such a conclusion by comparing the ratio between the diffusion coefficient of Ni in NiO and the self-diffusion coefficient of Ni (i.e., diffusion of Ni ions in Ni) (Figure 5f). Since this ratio is quite low compared to other metals, they concluded that the generation/migration rate of vacancies is well-balanced [32]. This led to the conclusion that when the generation and the migration rate of vacancies injected at the Ni/NiO interface as a consequence of the outward diffusion of Ni are comparable, the vacancies have the ability to migrate toward a position over a long-range distance where they aggregate and form a large single void instead of forming small voids distributed along the Ni/NiO interface. This effect was not observed with Cu because the generation rate of vacancies in Cu during oxidation was found to be much faster than the migration rate since the diffusion coefficient of Cu in Cu_2_O is much higher (up to nine times at 100 °C) than the self-diffusion coefficient of Cu. Thus, in the case of Cu, the generated vacancies do not have sufficient mobility to migrate over a long-range distance before they aggregate into one single large void. Rather, they form multiple small voids distributed along the Cu/Cu_2_O interface (as illustrated in Figure 5e), which leads to an additional step in the formation of a hollow Cu_2_O nanoparticle with a uniform shell thickness. The asymmetrical conversion mechanism identified when oxidizing spherical nickel nanoparticles was recently encountered by Railsback et al. who have shown that such an effect becomes more or less pronounced according to the size of the nanoparticles [25,43].

**Figure 6:**
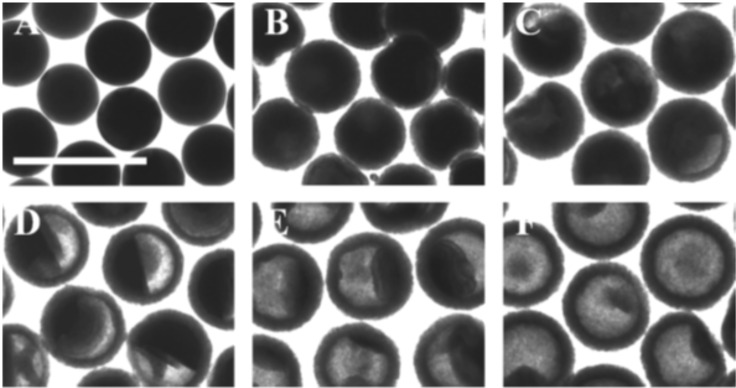
TEM micrographs showing the conversion stages of the CdS shell during the sulfidation of Cd nanospheres. The conversion process is monitored from the as-synthesized Cd nanospheres (A) until the fully hollow CdS nanospheres were obtained (B–F). Scale bar: 500 nm. Figure adapted with permission from [27], copyright 2008 American Chemical Society.

The formation of an off-centered, single void during the conversion process may also not be the only mechanism leading to the asymmetric shape of the hollow nanospheres. An example is the case of sulfidation of Cd nanospheres reported in the study by Cabot et al. who shown that, although an off-centered single void forms during the conversion process (Figure 6) [27], the spherical CdS shell grows isotropically resulting in the formation of hollow CdS nanospheres with a uniform shell thickness [27]. This puts in doubt the conclusions making a direct link between the formation of nonuniform shell thickness and the formation of a single void during the conversion of the particles. One obstacle preventing this phenomenon from being fully understood is related to the fact that the TEM observations are in general carried out ex situ. This means that the TEM micrographs collected during the different stages of the conversion process are not recorded on the same nanosphere. To provide a clearer interpretation of this phenomenon, direct monitoring of the conversion process using dynamic in situ TEM is mandatory.

#### Dynamic formation of hollow nanospheres

**Figure 7:**
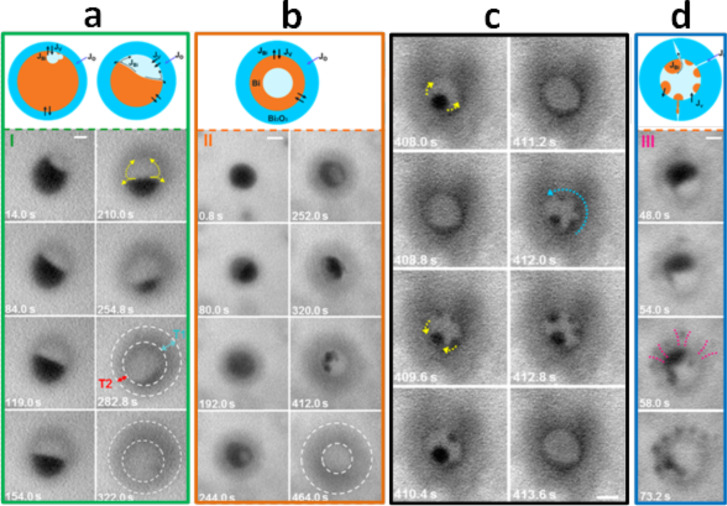
Sequential TEM micrographs recorded in situ to monitor the dynamic transformation of bismuth nanospheres during oxidation. The final product of the reaction was reported to be dependent on the considered nanosphere, which may transform by the end of the process into an: (a) asymmetric shell, (b, c) isotropic shell or (d) oxide shell decorated with bismuth nanoclusters. The data show that bismuth behaves like a liquid with frequent changes in the morphology of the nanodroplets inside the oxide shell. Scale bar: 50 nm. Figure adapted with permission from [48], copyright 2013 American Chemical Society.

Very recently, Niu et al. reported on the dynamic formation of bismuth oxide hollow nanospheres using a TEM liquid cell, allowing the in situ oxidization of bismuth nanoparticles to be observed [48]. Figure 7a presents a sequence of TEM micrographs recorded by the authors on a bismuth nanoparticle during oxidation. At the early stage, the oxidation process started with the formation of a thin layer of bismuth oxide on the outer skin of the bismuth nanoparticle (Figure 7a). An off-centered, single void was then formed at the bismuth/bismuth oxide core/shell interface. As the oxidation process proceeds in time, the void was found to become bigger while the bismuth core disappeared in a layer-by-layer fashion. By the end of the conversion process, a nonuniform oxide shell was formed. The authors demonstrated that this effect is related to the fact that bismuth diffuses out of the oxide shell nonuniformly with a faster diffusion rate through the shell adjacent to the void. In the same study, Niu et al. demonstrated that other particles oxidized under the same conditions do not show the same behavior (Figure 7b). Indeed, after the formation of an off-centered, single void, in the case of the second particle, they observed that the small amount of bismuth residue forming the core became unstable during the conversion process and behaves like a liquid rather than a solid. The bismuth core was found to suddenly split into several small nanodroplets (Figure 7b, 412 s) which then quickly vanish (Figure 7b, 464 s). The authors attributed the observed frequent changes of the bismuth configuration during oxidation to the local temperature which exceeds the melting point of the residual bismuth core evaluated in the same study of about 180 °C [48]. It is important to mention that this particle results in a uniform shell thickness at the end of the conversion process (Figure 7b, 464 s).

The liquid behavior of bismuth was also identified by the authors while imaging another particle (Figure 7c). They found that before achieving fully-hollow nanospheres, several unexpected events take place. Their data, presented in Figure 7c, show that after 408 s of oxidation, the bismuth nanoparticle becomes hollow with a bismuth nanodroplet on the curved inner surface of the shell. For an additional 0.8 s of oxidation, the formed bismuth nanodroplet wet the inner surface of the oxide shell. Then, when reaching 409.6 s, the authors observed that the nanodroplet tends to split into multiple tiny dewetting metal bismuth nanodroplets. The droplets then migrate and aggregate on the curved inner surface of the oxide shell before the bismuth wets the surface again (between 410.4 and 413.6 s). They attributed this reversible wetting transition to the interplay between the surface tension of the metal bismuth present in a liquid phase and the curved inner surface of the oxide shell. It is also believed that the wetting behavior of bismuth on the inner surface of the oxide shell can be influenced by other parameters such as the vapor pressure inside the oxide shell and the annealing temperature.

Niu et al. [48] have also remarked that in addition to the conversion mechanisms previously described, for some nanoparticles, bismuth nanoclusters can be formed on the outer surface of the particle (Figure 7d). They assumed that the defects present within the formed bismuth oxide shell (such as grain boundaries) serve as diffusion channels which enhance the outward diffusion of bismuth. They have further demonstrated that before being oxidized, bismuth tends to condense into several nanodroplets on the outer layer of the nanoparticle (Figure 7d, 73.2 s). In such a case, the growth rate of the oxide shell was found to be enhanced compared to the cases previously described.

### Nanotubes

#### Growth strategies

**Figure 8:**
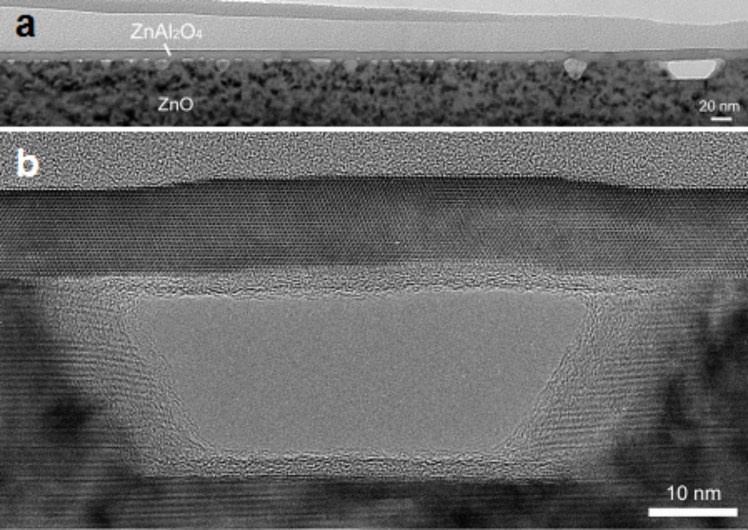
TEM (a) and high-resolution TEM (b) micrographs showing the Kirkendall voids formed at a ZnO–Al_2_O_3_ planar interface after annealing. Figure adapted with permission from [61], copyright 2007 American Chemical Society.

Following the report on hollow nanoparticles by Yin et al. in 2004 [7], Li and Penner [20] demonstrated in 2005 that the Kirkendall effect can be extended to the fabrication of nanotubes. CdS nanotubes were synthesized by high temperature sulfidation of Cd nanowires prepared by electrochemical step-edge decoration on graphite electrode surfaces. Since the as-grown nanowires were in contact with the surface of the graphite substrate, the diffusion of S was partially hindered, resulting in the formation of hemicylindrical CdS nanotubes instead of cylindrical ones. In 2006, Fan et al. [62] also showed that the Kirkendall effect can be used to synthesize monocrystalline spinel ZnAl_2_O_4_ nanotubes. In this case, the conversion method was based on a solid state reaction, occurring upon thermal annealing of core–shell ZnO/Al_2_O_3_ nanowires [23,62]. In such a process, the material forming the nanotube is defined by the two initial compounds constituting the core and the shell. As it can be seen in Figure 8, the formation of voids occurs at the interface within the ZnO which, in this case, plays the role of the fast diffusing material.

**Figure 9:**
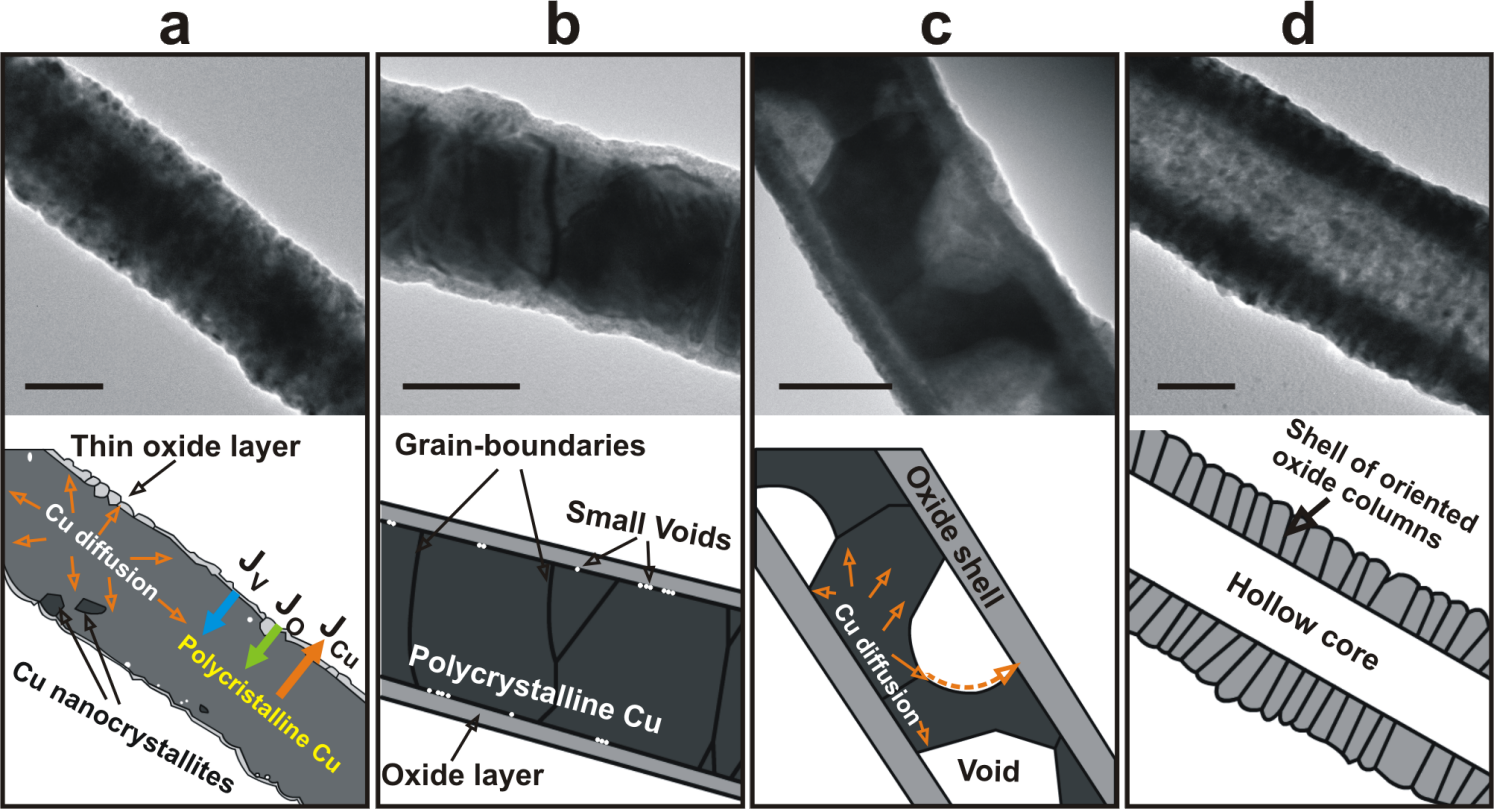
TEM micrographs and corresponding illustrations showing the chronological evolution of a Cu nanowire during thermal oxidation in air at 300 °C. The oxidation time was varied: (a) 1 min, (b) 2 min, (c) 3 min, and (d) 4 min. Scale bar: 100 nm. Figure adapted with permission from [63], copyright 2013 Wiley-VCH.

**Figure 10:**
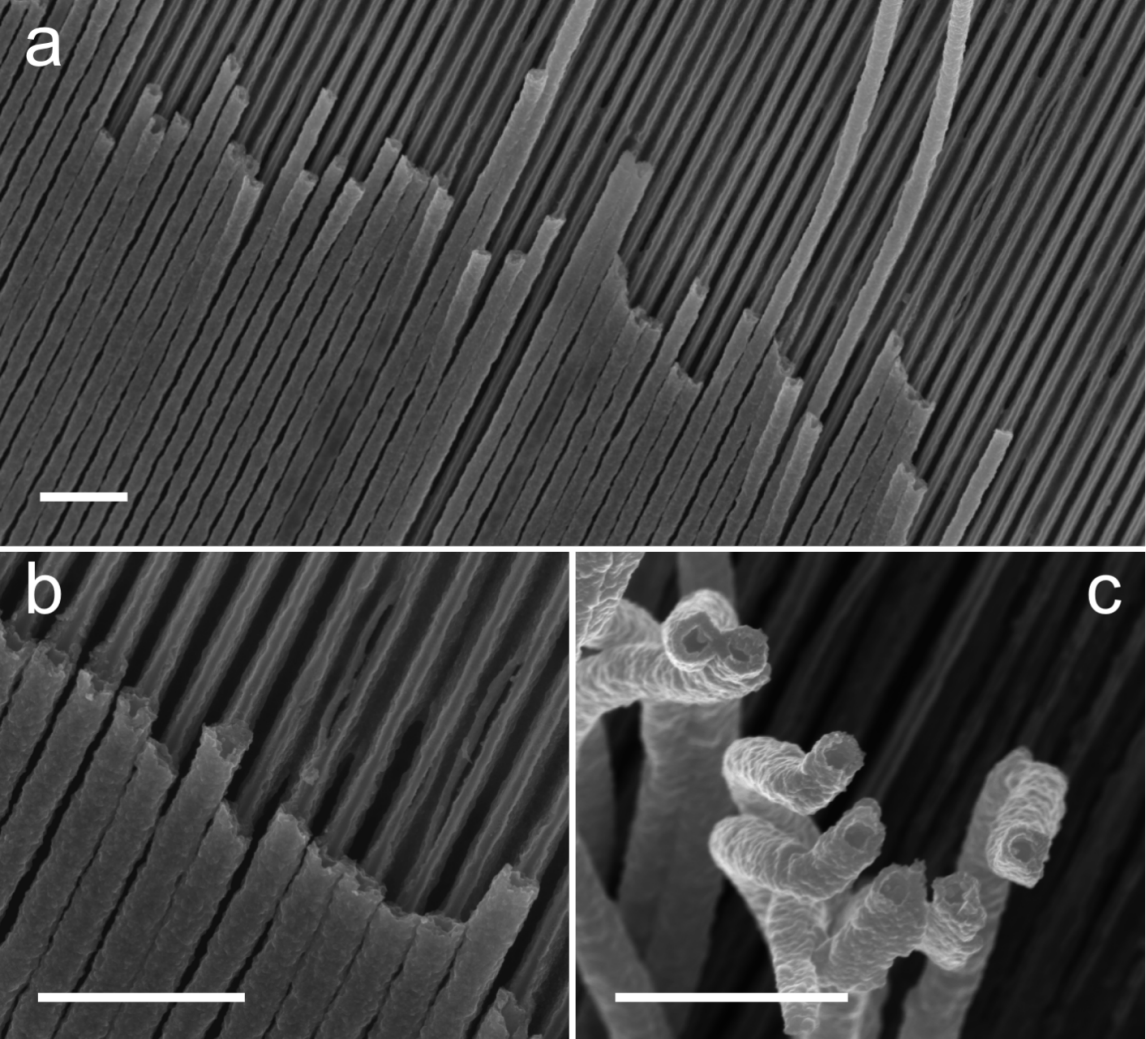
(a,b) SEM micrographs showing the high fidelity of copper oxide nanotubes prepared on nanograted silicon substrate by thermal oxidation of copper nanowires for 1 h at 300 °C. (c) SEM micrograph of copper oxide nanotubes disengaged from the nanograted substrate due to the cutting procedure applied to the specimen before observation with SEM. Scale bar: 1 μm. Figure adapted with permission from [63], copyright 2013 Wiley-VCH.

The strategy demonstrated by Li and Penner in 2005 was adopted by many groups for the synthesis of nanotubes by thermal annealing of metal nanowires under a controlled atmosphere such as sulfur, selenium or oxygen [15,63]. Among these materials, a preference was shown for metal oxide nanotubes due to the simplicity of oxidation of metals at ambient air conditions, as compared to sulfidation and selenization, where the use of toxic gases such as H_2_S and H_2_Se is required. On the basis of the large number of reports on this topic, similar to the case of the spherical nanoparticles presented in the previous section, the hollowing process of nanowires can now be easily described (Figure 9) [12,15,23,33,63–71]. Briefly, the conversion process starts with the adsorption of oxygen on the outer skin of the metal nanowire resulting in the formation of a thin layer of metal oxide (Figure 9a). After the formation of a metal/metal oxide core/shell nanowire, the metal ions diffuse outward through the oxide layer until reaching the outer surface. Simultaneously, the oxygen adsorbed on the outside of the wire diffuses through the oxide layer and penetrates toward the metal core. For some metals, such as Co, Cu, Fe, and Ni, the diffusion coefficients of metal ions through their own oxides are much higher than those of oxygen ions [34]. This fact results in the formation of multiple supersaturated vacancy clouds all over the metal/metal oxide interface along the wire axis which, in a further stage, condense and form multiple separated small voids within the metal core (Figure 9b,c) [63]. The coalescence of voids is the final mechanism occurring during the synthesis, which finally leads to the formation of hollow oxide nanostructures (Figure 9d and Figure 10).

#### Unusual effect in Kirkendall nanotubes

**Figure 11:**
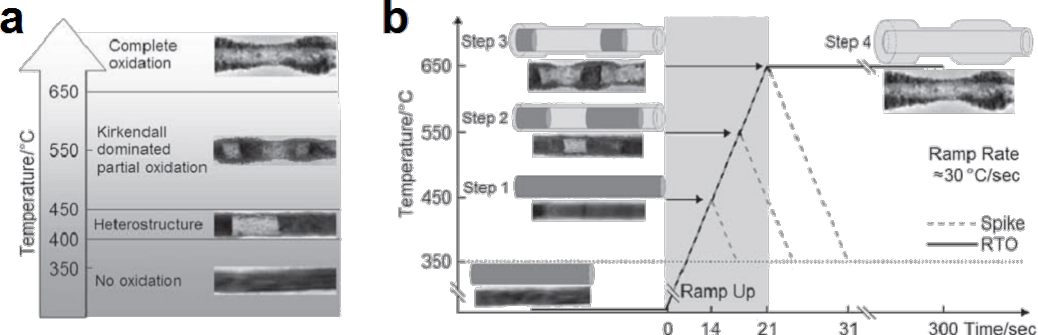
Representations of the different morphologies that can be obtained by thermal oxidation of nickel nanowires at different annealing temperatures and times. Two regimes are considered: (a) rapid thermal annealing and (b) ramp annealing. In the case of ramp annealing, the nanostructures in steps 1 to 3 are formed during the ramp up stage. The dashed lines in (b) represent the spike annealing temperature profiles. Figure adapted with permission from [66], copyright 2010 Wiley-VCH.

**Figure 12:**
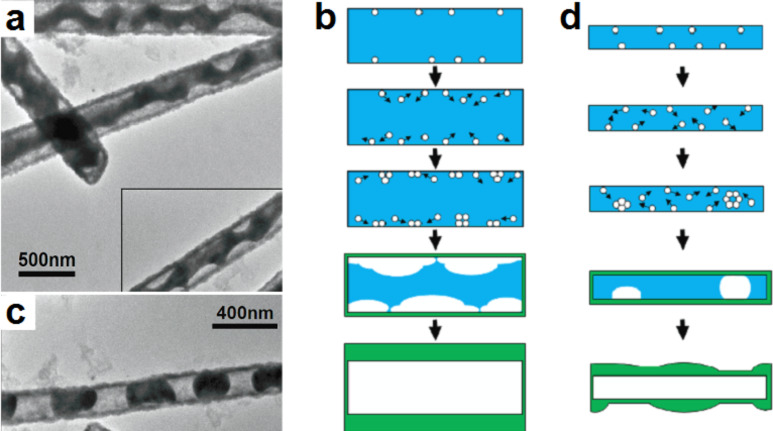
TEM micrograph of (a) thick (≈250 nm in diameter) and (c) thin (≈140 nm) nickel nanowires after thermal oxidation for 5 min at 500 °C in ambient air. Schematic showing the type of voids formed during oxidation of a nickel nanowire with a thick (b) and a thin (d) diameter. Figure adapted with permission from [67], copyright 2011 IOP Publishing.

Several reports have shown that the injection and the diffusion rates of vacancies play a key role in the Kirkendall-induced hollowing process of nanostructures [65–67]. In the case of oxidation of metal nanowires, the injection rate of vacancies can be defined as the difference between the outward and inward diffusion flux of the metal and oxygen ions through the oxide shell, respectively. The diffusion rate of vacancies within the metal is dependent on the self-diffusion rate of the metal ions. To create metal oxide nanotubes, two different annealing approaches can be used: (i) rapid thermal annealing [65] and (ii) gradient thermal annealing [66]. In general, the first approach is preferred over the second since the annealing time for a fixed temperature can be precisely controlled. For NiO nanotubes created by thermal oxidation of nickel nanowires, a systematic study was reported where both approaches were investigated (Figure 11) [66]. Through these studies, it was made clear that to obtain nanotubes with a uniform wall thickness, the diffusion rate of vacancies must be much lower than their injection rate [65–67]. When these conditions are not maintained, bamboo-like nanotubes may be formed [65–67]. This was first reported by Nakamura et al. while investigating the oxidation of nickel nanowires [65]. So far, this irregularity was only observed when oxidizing nickel nanowires. Although ramping (Figure 11a) and rapid (Figure 11b) thermal annealing approaches were investigated, the nanotubes were found to show the same bamboo-like structure at the final stage of the conversion process [66]. The formation of bamboo-like structures was attributed to the fact that nickel vacancies exhibit a very high diffusion length compared to other metals such as copper [65]. In a recent study, Ren et al. demonstrated that such irregularity originates from the fact that the diameter of the nickel nanowires used to create oxide nanotubes by means of the Kirkendall effect is in general much smaller than the diffusion length of nickel vacancies [67]. In their study, Ren et al. investigated the oxidation of nickel nanowires with two different diameters: ≈250 nm (Figure 12a,b) and ≈140 nm (Figure 12c,d) [67]. When the diameter of the nanowires is comparable to the vacancy diffusion length, the authors found that the vacancies were able to diffuse and agglomerate along the Ni/NiO interface (Figure 12a,b). As a consequence, multiple voids form along the Ni/NiO interface, which results in a further stage in the formation of nanotubes with uniform wall thickness. When the diameter of the nanowires becomes smaller or comparable to the diffusion length of vacancies in nickel, Ren et al. showed that a wall-to-wall diffusion can occur (Figure 12c,d) [67]. In other words, during oxidation, a vacancy generated at the interface on one side of the wire can travel across the wire and reach the opposite side before agglomerating with another vacancy (Figure 12d). This results in the formation of segmented-like nanotubes with a NiO shell and periodic nickel nanoparticles. As the oxidation process progress in time, the nickel atoms forming the nanoparticles undergo two diffusion mechanisms: lateral (perpendicular to the axis of the tube) and longitudinal (along the tube axis). Since no metal bridges are formed between the segmented metal particles, the diffusion along the tube axis was reported to be reduced and the lateral mechanism dominates. In the regions where the nanoparticles are formed, the Ni ions will diffuse laterally toward the outside of the nanotube. As this event occurs during oxidation, the segmented-like nanostructure transforms into a bamboo-like one.

**Figure 13:**
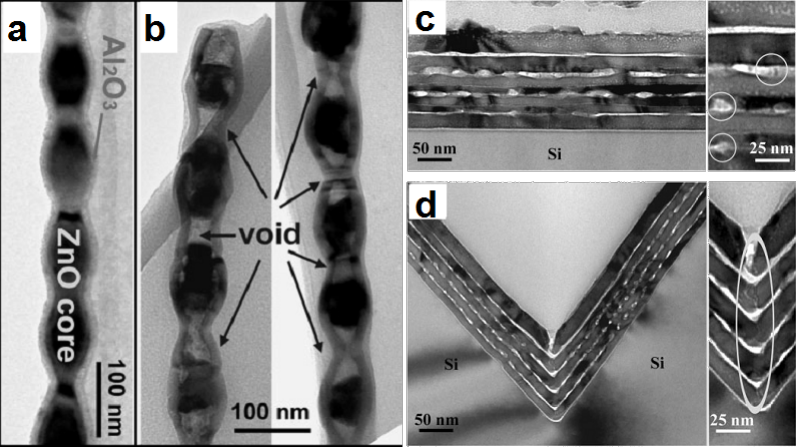
TEM micrographs of ZnO/Al_2_O_3_ core/shell oscillatory nanowires before (a) and after (b) annealing at 700 °C for 5 h. Cross-sectional TEM micrographs of ZnAl_2_O_4_ multilayers formed by a solid state reaction between ZnO and Al_2_O_3_ layers at 700 °C for 3 h in two different configurations: (c) planar (i.e., thin films) and (d) 3D (i.e., silicon V-trench). Figure adapted with permission from [72], copyright 2010 Wiley-VCH.

The presence of very high strain generated during oxidation due to the lattice mismatch between the metal core and the metal oxide shell is another factor that can amplify the formation of a bamboo-like structure. Güder et al. reported on a new approach based on stress engineering to control the spatial positioning and distribution of Kirkendall voids in ZnO/Al_2_O_3_ core–shell nanowires [72]. In this study, they applied their novel approach to synthesize ZnAl_2_O_4_ nanotubes containing ZnO nanocrystals. First, rippled ZnO nanowires coated with a 12 nm thick Al_2_O_3_ layer were synthesized (Figure 13a) and then annealed at 700 °C for 5 h (Figure 13b). The annealing process results in the transformation of the ZnO/Al_2_O_3_ nanowires into a peapod structure, where large nanocavities were formed in the thin areas (i.e., narrower section) of the wires, while the thick regions (i.e., wider sections) remained filled with ZnO (Figure 13b). The authors further demonstrate that such transformation is a consequence of the presence of an oscillating stress field with a periodic fluctuation along the wire’s axis due to the initial rippled structure of the core/shell nanowire [72]. Indeed, the fact that the stress gradient is higher in the thin areas of the wire compared to the thick ones, the void nucleation becomes more favorable in the narrower section of the wire compared to the rest of the regions. The theory proposed for core/shell ZnO/Al_2_O_3_ nanowires was further confirmed by the authors by annealing ZnO (24 nm)/Al_2_O_3_ (12 nm) periodic stacked layers. Two configurations were investigated: planar (Figure 13c) and 3D (V-trench substrate) (Figure 13d). In the planar configuration, disconnected Kirkendall channels/voids distributed without an ordered pattern were formed between the resulting ZnAl_2_O_4_ layers after annealing at 700 °C for 3 h (Figure 13c). On the other hand, the V-trench configuration shows a significant difference after annealing, where disordered smaller voids are distributed along the planar interface and large voids are pinned at the bottom of the V-trench (Figure 13d) [72]. From these results, the authors concluded that the formation of large voids at the bottom of the V-groove area is a consequence of the periodic stress pattern formed along the symmetry axis of the V-groove area of the deposited nanolayers, which results in the preferential nucleation and growth of voids.

## Conclusion

Recently, the Kirkendall effect has been considerably developed and used as a powerful process to synthesize hollow nanostructures of different shapes. We summarized the recent progress on the Kirkendall effect and discussed why it is considered as a versatile route to hollow nanostructures such as nanospheres and nanotubes. For nanospheres, we focused mainly on the comparison between the symmetrical and the asymmetrical conversion mechanisms that occur during the Kirkendall-induced hollowing process. The differences between these two mechanisms have been discussed in detail. In particular, we have discussed how the diffusion rate of vacancies as well as their injection rate can impact the final structure of the hollow nanospheres. Our discussion was further reinforced based on a newly published study reporting on the in situ dynamic TEM imaging of the Kirkendall-induced hollowing process of nanospheres.

For nanotubes, we have summarized the examples demonstrated so far using the Kirkendall effect. We dedicated special attention to oxide nanotubes. Based on the reports published since 2004, we discussed the hollowing mechanisms for one-dimensional materials and pointed out some unusual effects (i.e., formation of segmented and bamboo-like oxide nanotubes) observed in specific cases (i.e., oxidation of nickel nanowires) which, until today, is still not fully understood. The explanation reported so far to explain such unusual effects were discussed. More precisely, the presence of irregularities in nickel oxide nanotubes was related to the diffusion and to the injection rates of vacancies as well as to the confinement in nanowires.

Although huge efforts were dedicated to the comprehension of the fundamental mechanisms involved in the Kirkendall-induced hollowing process of nanomaterials, there are still several studies which must be carried out to expand the comprehension of this phenomenon. Among these, one can mention the study of the oxidation-induced Kirkendall conversion mechanisms of binary and ternary metal alloys. Such a study would be quite interesting since the presence of impurities in metals is expected to impact the diffusion coefficients, which might in turn influence the Kirkendall effect. Until today, only two studies were reported so far on this topic (i.e., oxidation of Ag/Au and Ni/Cr nanospheres) leaving a wide range of possible experiments which deserve to be conducted [73–74]. A very obvious study concerns the oxidation of binary and/or ternary metal alloy nanowires. This must provide additional elements for a better comprehension of the hollowing process since the vacancy confinement in 1D nanostructures differs from the case of nanospheres. Another perspective is the possibility of monitoring the oxidation-induced hollowing process using in situ TEM carried out under conditions similar to those applied ex situ (e.g., oxidation of nanostructures under an oxygen atmosphere at high pressure). Until now the dynamic studies were limited to the oxidation of nanostructures in TEM liquid cells [48]. However, such an experiment requires a very specific temperature-controlled TEM holder, allowing heating of the specimen at high temperature under an oxygen flow. Additionally, as far as we are aware, there are no comparative studies on the Kirkendall effect in monocrystalline and polycrystalline materials.

Another perspective which deserves to be pointed out is the TEM 3D tomography of Kirkendall nanospheres and nanotubes. This would allow a better comprehension of the hollowing mechanisms similar to some other processes such as the galvanic replacement, which has already been monitored using TEM tomography [75].

**Figure 14:**
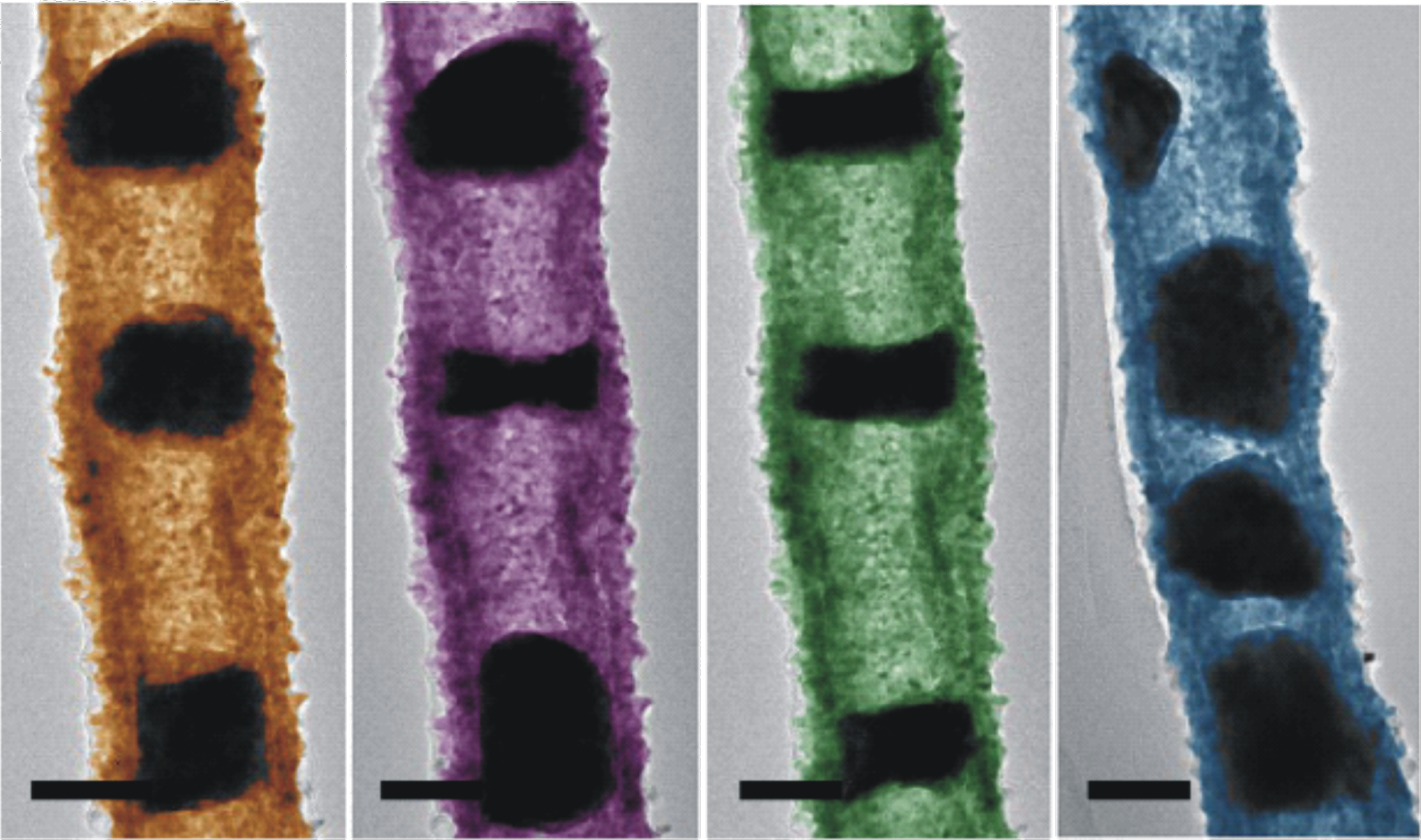
False-color TEM micrographs of some possible periodic Cu nanostructures that can be sculpted using an e-beam inside Kirkendall oxide nanotubes by local heating extracted from the source of a TEM. Scale bar: 100 nm. Figure adapted with permission from [64], copyright 2014 American Chemical Society.

In terms of fabrication, the Kirkendall effect may be combined with other fabrication processes. An example is to apply electron beam lithography to Kirkendall nanotubes, which allows the design of ordered periodic metal nanoparticles confined inside oxide nanotubes (Figure 14) [64]. Another possibility is the combination of the Kirkendall effect with galvanic replacement, which allows the fabrication of multilevel hollow nanostructures that otherwise cannot be prepared with one of these processes alone [76].

One of the important perspectives of this research topic is the understanding of the Kirkendall-induced hollowing process at the atomic level using computer modeling. It should be noted that a few models were developed so far [58,77–78], but they were limited to the modeling of the basic mechanisms of the hollowing process. Applying these models as well as dynamic simulation would be very helpful to explain the unusual phenomenon observed experimentally, such as the formation of asymmetric hollow nanospheres, segmented and bamboo-like nanotubes.
